# Supplementary material for: Anti-Ageing Potential of S. euboea Heldr. Phenolics
Source: Molecules. 2021 May 25;26(11):3151. doi: 10.3390/molecules26113151 (PMC8198620; doi:10.3390/molecules26113151)
Supplement: Supplementary file 1 [file molecules-26-03151-s001.zip › molecules-1187203-SI.pdf]

molecules

MDPI

Supplementary Materials

Anti-Ageing Potential of *S. euboea* Heldr. Phenolics

Ekaterina-Michaela Tomou [1], Christina D. Papaemmanouil [2], Dimitrios A. Diamantis [2], Androniki Kostagianni [2], Paschalina Chatzopoulou [3], Thomas Mavromoustakos [4], Andreas G. Tzakos [2,5,*] and Helen Skaltsa [1,*]

[1] Department of Pharmacognosy & Chemistry of Natural Products, School of Pharmacy, National and Kapodistrian University of Athens, Panepistimiopolis, Zografou, 15771, Athens, Greece; ktomou@pharm.uoa.gr (E.-M.T.), skaltsa@pharm.uoa.gr (H.S.)

[2] Department of Chemistry, Section of Organic Chemistry and Biochemistry, University of Ioannina, 45110 Ioannina, Greece; christina.pa@hotmail.gr (C.D.P.), dimitrisdiamantis0@gmail.com (D.A.D.), androniki-kostagianni@gmail.com (A.K.), atzakos@uoi.gr (A.G.T.)

[3] Hellenic Agricultural Organization DEMETER, Institute of Breeding and Plant Genetic Resources, IBPGR, Department of Medicinal and Aromatic Plants, Thermi, 57001 Thessaloniki, Greece; xatzlin@yahoo.gr (P.C.)

[4] Department of Chemistry, National and Kapodistrian University of Athens, Panepistimiopolis, Zografou 15771, Greece; tmavrom@chem.uoa.gr (T.M.)

[5] University Research Center of Ioannina (URCI), Institute of Materials Science and Computing, Ioannina, Greece; atzakos@uoi.gr (A.G.T.)

* Correspondence: skaltsa@pharm.uoa.gr (H.S.); atzakos@uoi.gr (A.G.T.)

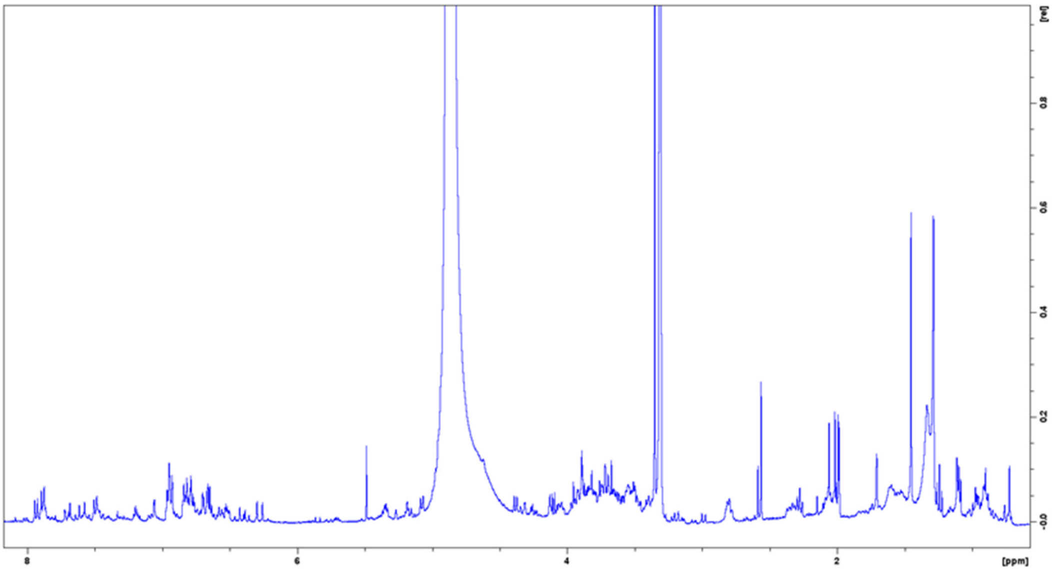

Figure S1. ^{1}H-NMR spectrum of EtOAc residue of cultivated *S. euboea* (CD_3OD, 400 MHz) (T=295 K; number of scans, 16).

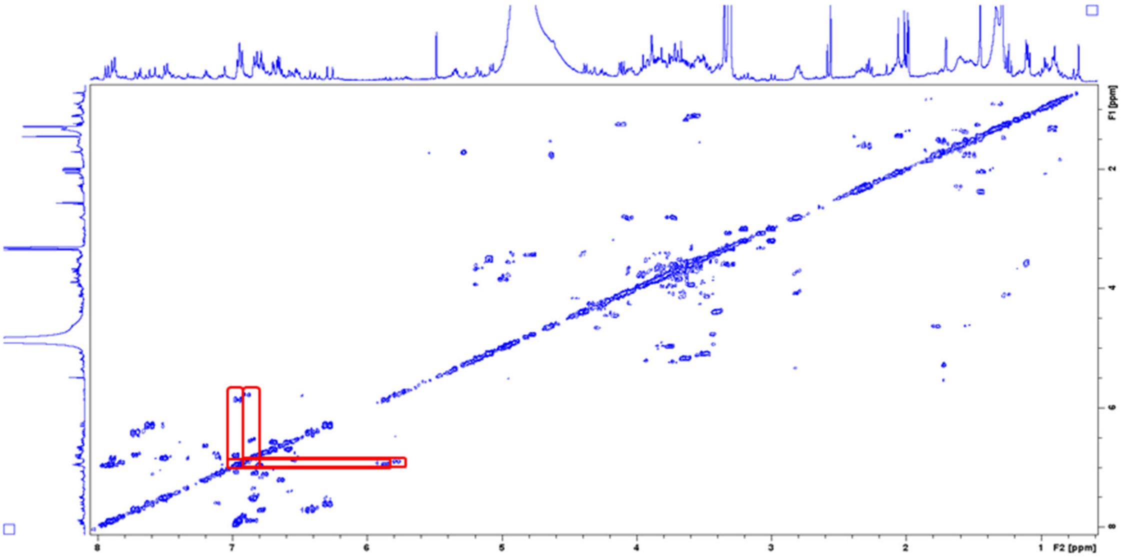

Figure S2. COSY spectrum of EtOAc residue of cultivated *S. euboea* (CD_3OD, 400 MHz) (T=295 K; number of scans, 4).

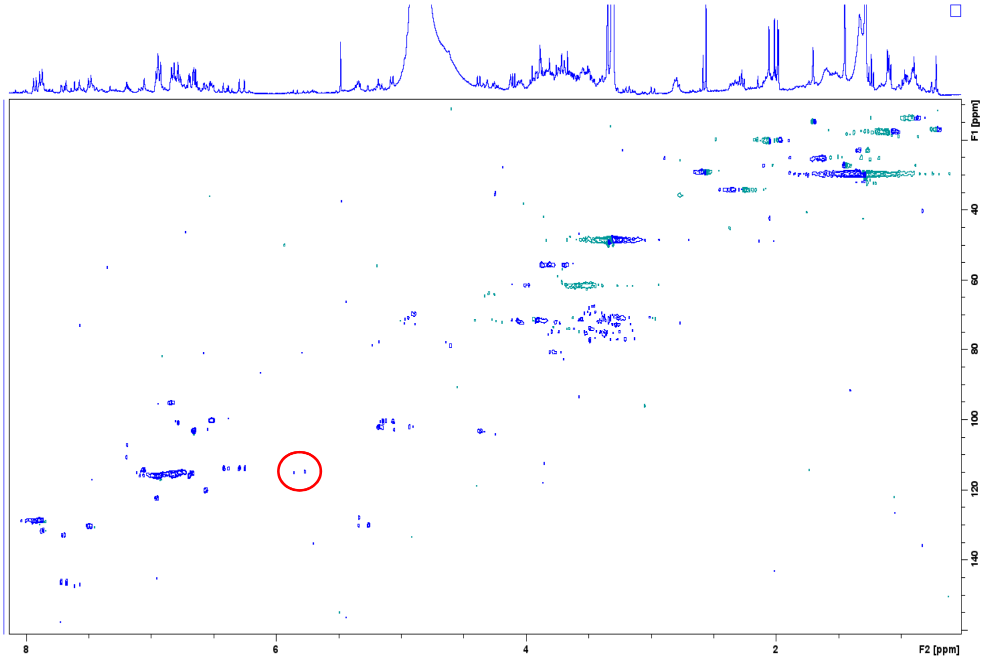

Figure S3. HSQC spectrum of EtOAc residue of cultivated *S. euboea* (CD_3OD, 400 MHz) (T=295 K; number of scans, 22).

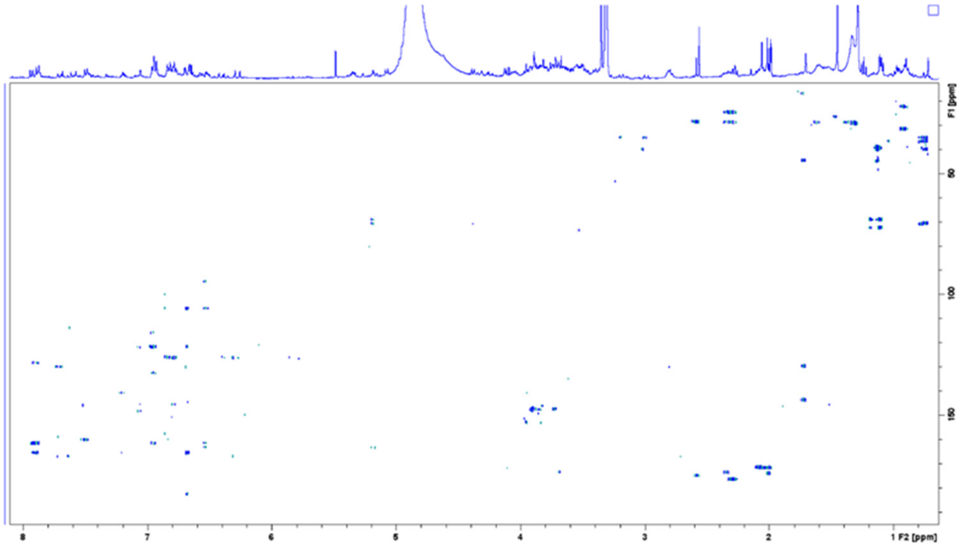

Figure S4. HMBC spectrum of EtOAc residue of cultivated *S. euboea* (CD_3OD, 400 MHz) (T=295 K; number of scans, 22).

Table S1. Chemical structures of the isolated compounds **1-33.**

**(1)**	**(2)**	**(3)**

	R_1	R_2	R_3	R_4	
**(4)**	$COOCH_3$	H	OH	H	
**(5)**	H	OH	H	OAc	**(6)**

	R_1	R_2	R_3	R_4	R_5	R_6
**(7)**	OH	H	OH	H	H	OH
**(8)**	H	H	OH	H	H	OH
**(9)**	H	H	*O*-glu	H	H	OH
**(10)**	H	H	3''-*O*-*trans*-p-coumaroyl-glu	H	H	OH
**(11)**	H	H	4''-*O*-*trans*-p-coumaroyl-glu	H	H	OH
**(12)**	H	H	4''-*O*-*cis*-p-coumaroyl-glu	H	H	OH
**(13)**	H	H	6''-*O*-*trans*-p-coumaroyl-glu	H	H	OH
**(14)**	H	H	6'''-*O*-acetyl-allosyl]-(1→2)-glu	OH	H	OH
**(15)**	H	H	6'''-*O*-acetyl-allosyl]-(1→2)-6''-*O*-acetyl-glu	OH	H	OH

(**16**)	H	H	6'''-*O*-acetyl-allosyl]-(1→2)-glu	OH	OH	OH
(**17**)	H	H	6'''-*O*-acetyl-allosyl]-(1→2)-glu	OH	OH	OCH_3
(**18**)	H	H	6'''-*O*-acetyl-allosyl]-(1→2)-6''-*O*-acetyl-glu	OH	OH	OCH_3

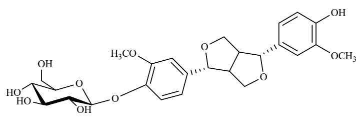
(**19**)

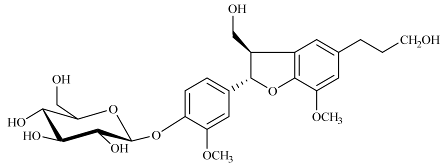
(**20**)

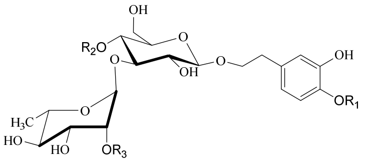

	R₁	R₂	R₃
(**21**)	H	*trans*-caffeoyl	H
(**22**)	H	*cis*-caffeoyl	H
(**23**)	H	*trans*-feruloyl	H
(**24**)	H	*cis*-feruloyl	H
(**25**)	CH_3	*trans*-feruloyl	H
(**26**)	H	*trans*-caffeoyl	Gal

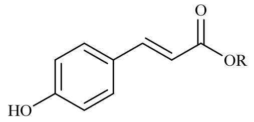

(**27**)	R=H
(**28**)	R=CH_3

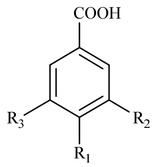

	R₁	R₂	R₃
(**29**)	OCH_3	H	H
(**30**)	OH	H	H
(**31**)	OH	OCH_3	H
(**32**)	OH	OCH_3	OCH_3

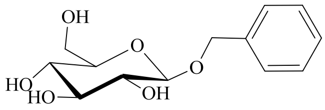
(**33**)

Glu: Glucopyranoside; Gal: Galactopyranoside.

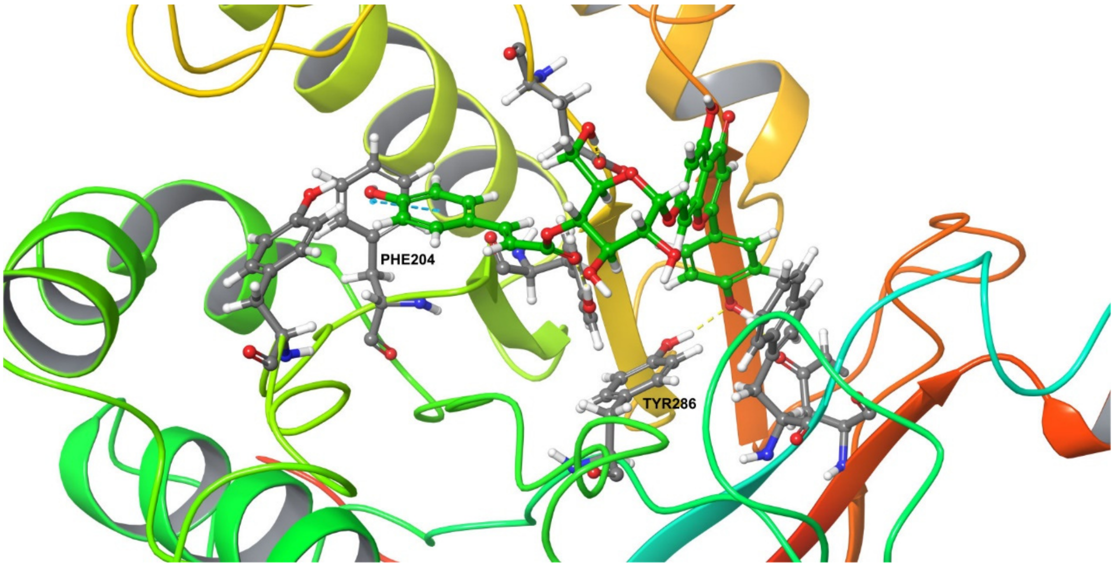

Figure S5. Best pose of the interaction of **compound 12** with hyaluronidase. The pi-stacking interactions are shown with light blue dashed lines and the hydrogen bonds with yellow dashed lines.

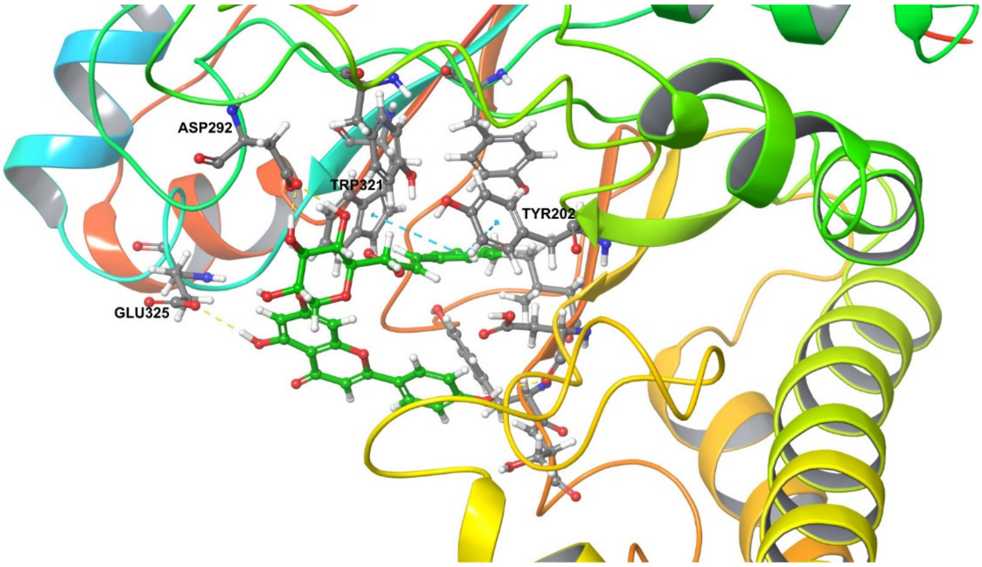

Figure S6. Best pose of the interaction of **compound 13** with hyaluronidase. The pi-stacking interactions are shown with light blue dashed lines and the hydrogen bonds with yellow dashed lines.

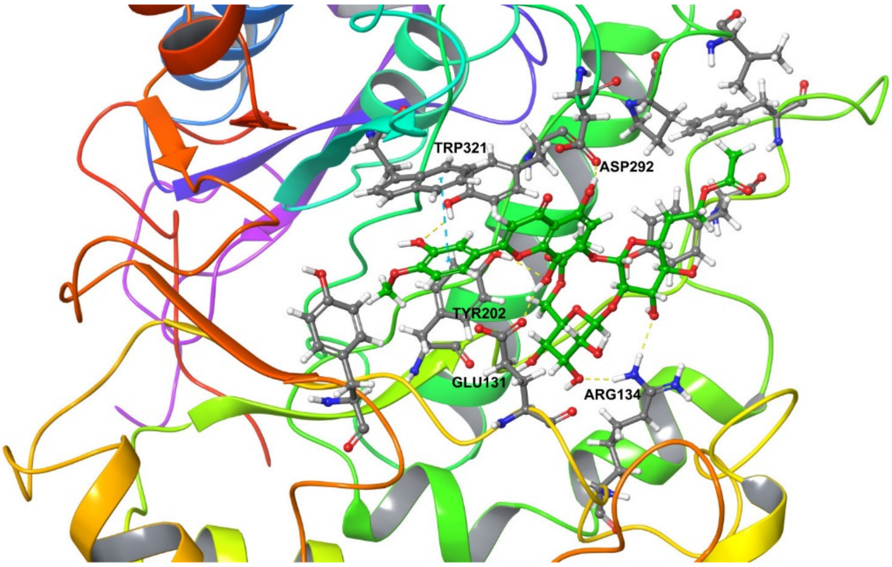

Figure S7. Best pose of the interaction of **compound 17** with hyaluronidase. The pi-stacking interactions are shown with light blue dashed lines and the hydrogen bonds with yellow dashed lines.

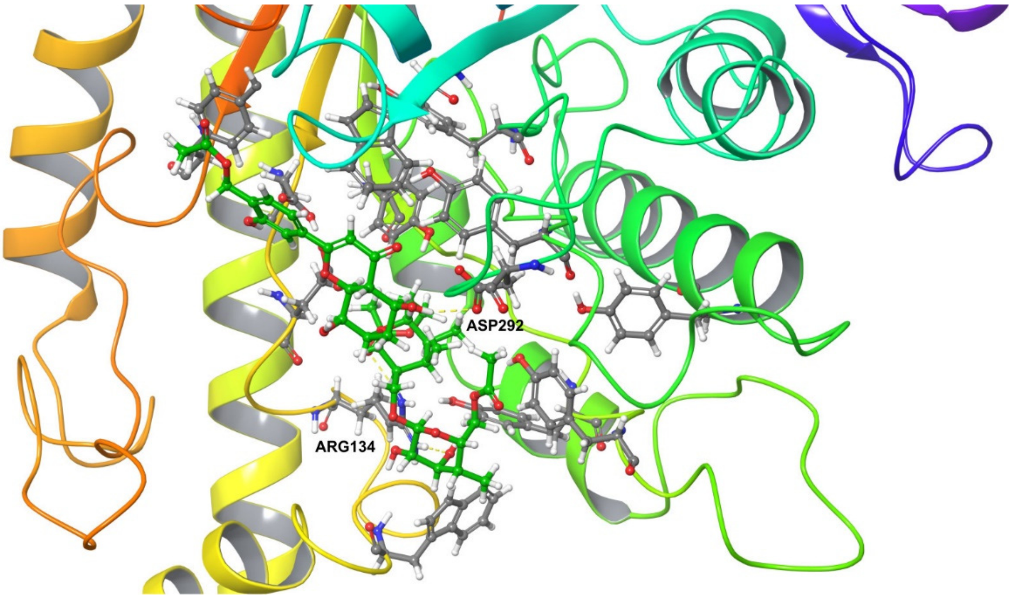

Figure S8. Best pose of the interaction of **compound 17** with hyaluronidase. The pi-stacking interactions are shown with light blue dashed lines and the hydrogen bonds with yellow dashed lines.

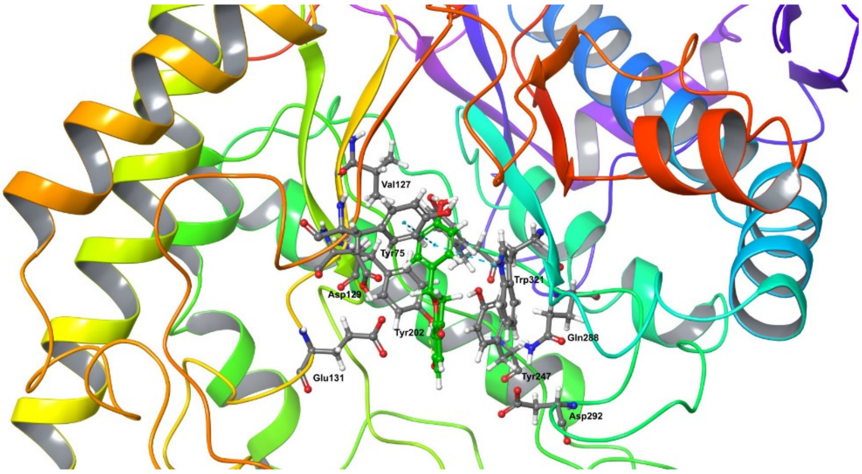

Figure S9. Best pose of the interaction of **liquiritigenin** with hyaluronidase, via Maestro software, showing liquiritigenin inside the cavity oh hyaluronidase interacting with the amino acid residues of the active site.

According to both pieces of software, liquiritigenin was located in the active site of hyaluronidase surrounded by the amino acid residues Tyr75, Val127, Tyr202, Tyr247, Tyr286, Trp321, Asp129, Glu131, Gln288 and Asp292. Comparing the known inhibitor, liquiritigenin, to our studied isolated compounds, it is shown that the latter displayed significant similarity regarding the interaction profile. As described in our manuscript, compound **9** interacted with the same active site amino acid residues Tyr75, Tyr202, Tyr247, Tyr286, Trp321, Glu131, and Asp292 (**Figure 6**).
